# The association of access to green space with low mental distress and general health in older adults: a cross-sectional study

**DOI:** 10.1186/s12877-024-04738-3

**Published:** 2024-04-10

**Authors:** Heidi Lyshol, Rune Johansen

**Affiliations:** 1 Department of Health and Inequality, Norwegian Institute of Public Health, Oslo, Norway; 2 Department of Mental Health and Suicide, Norwegian Institute of Public Health, Oslo, Norway

**Keywords:** General health, Mental distress, Green space, Older adults

## Abstract

**Background:**

Access to green space is considered beneficial to mental and physical health, though the causal pathways are not completely clear. Accordingly, the objective of this study was to examine how access to green space was associated with low mental distress and general health among older adults.

**Methods:**

The data in our study stems from a survey from three Norwegian counties in 2015-16, *n* = 2937. The main exposure variable was self-reported access to green space, and the outcome variables were self-reported mental distress and general health. Logistic regression adjusted for sex, age, education, physical activity, functional disability, access to cultural/sports facilities and economic security was performed to assess the association between the exposure variable and the outcome variables.

**Results:**

Access to green space was associated with both higher odds of low mental distress (Odds Ratio = 3.85**, 95% CI 2.04–6.02) and good general health (OR = 8.20**, 95%CI 5.88–11.49) compared to no access. In models adjusted for sex, age, and education, the ORs were only slightly changed (OR = 4.03**, 95%CI 2.52–6.45) and (OR = 7.91**, 95%CI 5.63–11.13). However, adjusting for general health with low mental distress as outcome, the association was no longer statistically significant; (OR = 1.28 95%CI 0.74–2.21). Adjusting for low mental distress with general health as outcome, the association remained statistically significant; (OR = 3.43** 95%CI 2.34–5.03).

**Conclusions:**

Our findings suggest that the association between access to green space and mental health may be mediated by general health. This implies that studies of associations between access to green space and mental health must take general health into consideration.

**Supplementary Information:**

The online version contains supplementary material available at 10.1186/s12877-024-04738-3.

## Background

The global population is ageing and becoming more urban [1]. With age comes more health challenges, many of them associated with a sedentary lifestyle [2]. Wishing to live in urban surroundings is also consistent with the wishes of the ageing Norwegian population [3], but urban surroundings do not imply that people have to be sedentary. To ensure a good future for older adults and other vulnerable populations, there is a need to plan for an environment that makes it easy to maintain physical and mental health [2–5]. Green spaces, including both urban parks, nature paths and forests and other natural environments, are considered to be particularly positive for the health of the elderly [4]. Reasons for this may be manyfold. Proximity may lead to increased use, plant life has a beneficial effect on air quality, and studies have also shown positive mental effects of being in natural surroundings [1, 6–8].

Physical activity is associated with increased quality of life for older adults and seems to prevent age-related deterioration in both physical and mental health [9–11]. In Norway, physical activity in green spaces, known as *friluftsli*v (“outdoor life”) [12, 13] a concept introduced by famous playwright Henrik Ibsen [14], is a national ideal, and older adults are recommended to be active outdoors [15]. This emphasis on physical activity outdoors is also mirrored in the Norwegian interministerial action plan “Sammen om aktive liv” (“Together for active lives”) [2, 16]. Studies have emphasized the value of outdoor activity for physical and mental health in older adults [17–21]. Markevych et al. [22], Lachowycz & Jones [23] and Maas et al. [24] have discussed possible mechanisms around access to green space and health, stressing the lack of data. Similarly, there seem to be few studies using data on self-reported access to green space and physical activity in relation to both mental and general health in Norway.

Astell-Burt et al. [25] have demonstrated a pathway from access to green space via physical activity to good mental health in a large study from Australia, which is not supported by the findings of Maas et al. in the Netherlands [24]. We were curious about whether this could be related to the geographical or cultural differences between Australia and the Netherlands and wanted to see what our Norwegian data set would show.

Ohrnberger et al. [26] have discussed the pathways between physical and mental health, including a theoretical model for mediation that includes physical activity, but they do not focus on access to green space. We were particularly interested in access to green space and used this as starting point for our analyses.

Health is associated with behaviours, but also with structural and social factors [27, 28]. We were therefore also interested in seeing how other structural factors, such as access to cultural and sports venues, behavioural factors, such as physical activity, and social factors, such as economic security, might affect the associations between access to green space and health outcomes.

### Objectives

The aims of the present study were to assess the associations between the structural factor self-reported access to green space and general health and low mental distress in older adults.

## Materials and methods

We used data about mental distress, general health, physical activity, access to green space, access to cultural/sports venues, functional disability, and economic security from a regional cross-sectional health interview survey, which was conducted in 2015-16 in the Norwegian counties Vest-Agder, Aust-Agder and Vestfold on the request of the Norwegian Ministry of Health as a pilot for further county surveys [27]. The participants lived in cities with up to 90 000 inhabitants as well as in more rural areas. Information about age and sex were linked from the national population register by the unique 11-digit personal identification number assigned to every resident of Norway, while education was linked from the national education register.

### Participants

The sample included in our study was 5021 randomly selected adults aged 65–100 years living at home. The survey allowed for various ways of responding. At first, invited individuals had the option to answer by post or web. After two weeks, non-respondents were contacted by phone for an interview. In our study population, the response rate was 58%; 1413 men and 1524 women. Out of these, 1064 men and 1004 women responded to all the questions and were included in our analyses. (See also Supplementary Table A).

### Outcomes

The short five-item version of the Hopkins Symptom Check List (HSCL-5) [28] was used as a measure for mental distress. All five questions were scored on a four-category scale: from no to severe symptoms, with a total score between 5 and 20, thus yielding a mean score between 1 and 4. A mean value of 2.0 or higher is found to be indicative of mental distress [28], and this cut-off point was used to dichotomize the variable into high and low mental distress.

Self-reported general health was assessed by the question “How do you rate your health in general?”, with five possible responses: ‘very good’, ‘good’, ‘fair’, ‘poor’, and ‘very poor’ [29]. The variable was dichotomized by collapsing the categories ‘very good’ and ‘good’ defined as good, and ‘fair’, ‘poor’, and ‘very poor’ defined as poor. This question is a highly validated and reliable instrument on general health, which primarily measures physical health. It has been in use for decades [30].

### Main exposure

Access to green space was assessed by the question “Do you feel that it is easy for you to get to nature and recreation areas?” (yes/no). We must emphasize that this is a subjective, self-reported measure, not based on objective geographical information.

### Confounders

Age was grouped into the categories 65–74, and 75 + years. Highest achieved education levels from the Education registry were grouped into two categories, not finished secondary school or less (≤ 13 years of schooling; ISCED categories 0–3) and finished secondary school and higher; ≥14 years of schooling; ISCED categories 4–7) [31].

Physical activity was assessed by the question «How often do you exercise every week, outside of work?», as used by the Eurostat EHIS surveys [32], but with the following six answering categories; “Never”, “Less than weekly”, “Once a week”, “2–3 times per week”, “4–5 times per week”, “Almost daily”. The definition of exercise/physical activity included moderate activities, such as walking or gardening. The indicator was dichotomized, with those exercising weekly or more often in the higher category, and those exercising less than once a week or never in the lower category. This confounder variable was also regarded as a predictor in some of the analyses, because much of the literature stressed the particular importance of physical activity in the interplay between access to green space and mental or physical health.

Functional disability was assessed with the question: “Do you have any functional disabilities or afflictions caused by injuries, including intermittent problems?” (yes/no), based on the set of EHIS questions [32]. This variable was included in our analyses because such disabilities may affect both how the respondent considered their access to green space and also whether physical activity was possible.

Access to culture/sports venues was assessed by the question “Do you feel you have easy access to culture/sports facilities?” (yes/no). This variable was included because using such facilities might constitute an alternative to using green space.

Economic security was assessed by the question “Could your household afford to pay an unexpected bill of 10 000 NOK (about 1000 €) without having to take out a loan or receive financial help?” (yes/no). This variable was included because economic conditions might influence to which degree people might feel free to partake in “friluftsliv”.

We have reversed the outcome mental distress and the indicator functional disability into low mental distress and having no functional disability because they point in the opposite direction of all the other indicators and the general health outcome. This made it challenging to present the analyses in a consistent manner. Reversing these variables makes the presentations more understandable. Now lack of functional disability and low mental distress are considered positive or protective, and high values of these, like for all the other factors in our analyses, are associated with (good) general health. See Tables 1 and 2.

**Table 1:** Overview of the variables

	n_detrimental_ (%)	n_positive_ (%)
Mental distress (yes/no)	112 (5.4%)	1 956 (94.6%)
General health (poor/good)	584 (28.2%)	1 484 (71.8%)
Access to green space (no/yes)	192 (9.3%)	1 876 (90.7%)
Physical activity (less than weekly/weekly or more often)	320 (15.5%)	1 748 (84.5%)
Access to culture/sports venues (no/yes)	208 (10.1%)	1 860 (89.9%)
Functional disability (yes/no)	823 (39.8%)	1 245 (60.2%)
Economic security (no/yes)	145 (7.0%)	1 923 (93.0%)
**Other variables**	**n (%)**	**n (%)**
Age (65–74/75+)	1 395 (67.5%)	673(32.5%)
Sex (men/women)	1 064 (51.5%)	1 004 (48.5%)
Education (secondary school or not) (no/yes)	1 034 (50.0%)	1 034 (50.0%)

N = 2068, detrimental categories in left column for the relevant variables

**Table 2:** Cross-tabulations between the health outcomes and the other variables

		General health		HSCL-5 score	
		Bad	Good	Low	High
Education	< Secondary school	367	667 (64.5%)	966	68 (6.6%)
	Secondary school	217	817 (79.0%)	990	44 (4.3%)
Age group	65–74	372	1023 (73.2%)	1316	79 (5.7%)
	75+	212	461 (68.5%)	640	33 (4.9%)
Sex	Men	286	778 (73.1%)	1011	53 (5.0%)
	Women	298	706 (70.3%)	945	59 (5.9%)
Access to culture/sports	No	102	106 (51.0%)	181	27 (13.0%)
	Yes	482	1378 (74.1%)	1775	85 (4.6%)
Access to green space	No	138	54 (28.1%)	163	29 (15.1%)
	Yes	448	1430 (76.2%)	1793	83 (4.4%)
Physical activity	<Weekly	168	152 (47.5%)	283	37 (11.6%)
	Weekly+	416	1332 (76.2%)	1673	75 (4.3%)
Economic security	No	74	71 (49.0%)	120	25 (17.2%)
	Yes	510	1413 (73.5%)	1836	87 (4.5%)
(lack of) Functional disability	No	408	415 (50.4%)	748	75 (9.1%)
	Yes	176	1069 (85.9%)	1208	37 (3.0%)

### Statistical analyses

As the main goal was to explore the associations between access to green space and low mental distress and good general health, we analysed the models with high and low mental distress and good and poor general health as outcomes, and access to green space as exposure using SPSS 27. Age, sex, education, physical activity, (lack of) functional disability, access to culture/sports venues, and economic security were considered as covariates. All significance is at a 0.05% level unless otherwise stated.

In the main analyses, univariate and multiple logistic regression were used to assess the associations between the main exposure, access to green space, and the health outcomes, adjusting for the confounders. In the univariate analyses (model 0), the outcomes were analysed successively with respect to the other variables, both the main exposure and the confounders, one by one. Model 1 was like model 0, with additional adjustments for age, sex, and education. In model 2, the main exposure and the other independent variable, physical activity, and the confounders, functional disability, access to cultural/sports facilities and economic security, were included, excluding the other health outcome (as an independent variable). Finally, in model 3, the analyses in model 2 were repeated, adding the other health outcome as an independent variable.

Linear models were considered, but ultimately relegated to the Supplementary materials (Supplementary Tables B1-B4), due to the nature of the variables. These variables are diverse, both dichotomous and continuous, and several of them have a skewed distribution. Linear regression models are based on normally distributed data. With our material, a linear regression would not necessarily give clear results. Although information may be lost in dichotomization, this was seen as necessary to provide results that would stand up to interpretation. Linear regression was performed for the purpose of consistency control. In these linear models we used the disaggregated versions of the confounders: all the available ISCED categories for education, all the categories for physical activity and 1-year age categories. Potential interaction effects between the main exposure, access to green space, and the various confounders were also included in the linear analyses.

## Results

In our sample of 2068 older adults, poor general health was reported by 28.2%, while 5.4% reported mental distress. 50.0% had finished secondary education or more. 84.5% were physically active at least once a week. Access to culture/sports venues was reported by 89.9%, and access to green space was reported by 90.7%, while 93.0% were economically secure. 1064 were men, 1004 were women, while 1395 were in the age group 65 − 64, 673 were 75 and older. The oldest participant was 100 years old.

We have chosen to include the covariate physical activity in Tables 3 and 4 because much of the literature shows that the associations between access to green space and health outcomes may be related to physical activity, leaving this particular confounder in a special position as an independent variable. They are also correlated, the Pearson coefficient and Spearman’s rho both being 0.24**. The other confounders are excluded from Tables 3 and 4.

**Table 3:** Logistic regression with low mental distress as dependent variable, with independent variables access to green space, physical activity, and good general health n = 2068^§^

Predictor variable	Model 0	Model 1	Model 2	Model 3
Access to green space	3.85** [2.04?6.02]	4.03** [2.52?6.45]	1.81* [1.06?3.11]	1.28 [0.74?2.21]
Physical activity	2.94** [1.93?4.41]	2.56** [1.70?4.29]	1.93** [1.23?3.02]	1.51 [0.96?2.39]
Good general health	8.55** [5.75?12.66]	7.87** [5.08?12.20]	-	5.13** [3.13?8.40]

§ Odds ratios (OR) with 95% confidence intervals in brackets

** p<0.01, * p<0.05

Model 0: Unadjusted univariate OR

Model 1: Model 0, adjusted for sex, age, and education

Model 2: Multivariate model including all confounders and excluding general health

Model 3: Multivariate model including all confounders and including general health

**Table 4:** Logistic regression with good general health as dependent variable, with independent variables access to green space, physical activity, and low mental distress n = 2068^§^

Predictor variable	Model 0	Model 1	Model 2	Model 3
Access to green space	8.20** [5.88-11.49]	7.91** [5.63-11.13]	3.51** [2.41-5.11]	3.43** [2.34-5.03]
Physical activity	3.53** [2.63-4.52]	3.27** [2.55-4.20]	2.53** [1.91-3.37]	2.47** [1.90-3.38]
Low mental distress	8.55** [5.75-12.66]	7.87** [5.08-12.20]	-	5.29** [3.23-8.62]

§ Odds ratios (OR) with 95% confidence intervals in brackets

** p<0.01, * p<0.05

Model 0: Unadjusted univariate OR

Model 1: Model 0, adjusted for sex, age, and education

Model 2: Multivariate model including all confounders and excluding low mental distress

Model 3: Multivariate model including all confounders and including low mental distress

Both the exposure, access to green space, and the other variable of particular interest, physical activity, were significantly associated (*p* < 0.01) with both good general health and low mental distress in both Model 0 and Model 1 (Tables 3 and 4). Moreover, the odds ratios were only slightly attenuated when adjusting for age, sex, education, access to cultural or sports venues, economic security and (lack of) functional disability.

Using low mental distress as outcome and excluding general health as an independent variable, the OR of access to green space was 1.81*, 95% CI=[1.06–3.11] (Table 3, Model 2). After adjusting for general health, the OR of access to green space was 1.28, 95% CI=[0.74–2.21] (Table 3, Model 3). The OR of general health was 5.13**, 95% CI=[3.13–8.40]. Adjusting for general health, the association between access to green space and mental health drops from 1.81 to 1.28 and is no longer statistically significant.

Considering general health as outcome, excluding mental distress as an independent variable, yielded an OR of access to green space of 3.51**, 95% CI=[2.41–5.11] (Table 4, Model 2). After adjusting for mental distress, the odds ratio of access to green space changed slightly to 3.43**, 95% CI=[2.34–5.03] (Table 4, Model 3). The OR of low mental distress was 5.29**, 95% CI=[3.23–8.62].

That the change in OR is so small, from 3.51 to 3.43, and both ORs remain statistically significant, suggests that mental health only to a small degree affects the association between access to green space and physical health.

In the linear models (Supplementary Tables B1-B4), we saw the same patterns as in the logistic models, thus confirming that using the latter was an acceptable choice. Supplementary Tables B1-B2 show that there was an interaction between access to green space and physical activity when low mental distress was the outcome. However, Supplementary Tables B3-B4 show no such interaction for the outcome general health. In the binary logistic models, we found no statistically significant interaction effects between access to green space on the one hand, and physical activity, age, gender, and education on the other hand.

## Discussion

### General considerations on mediation

According to Baron & Kenny [33], a variable functions as a mediator when it meets the following conditions;

1. Variations in levels of the predictor significantly account for variation in the presumed mediator,
2. Variations in the mediator significantly account for variations in the outcome,
3. When (1) and (2) are controlled for, a previous significant relation between the predictor and outcome is no longer statistically significant.

Since it is plausible that access to green space influences health positively [34], and good general health is associated with low mental distress [35], and much of the association between access to green space and mental health disappears when controlling for general health, the results above may suggest that general health mediates the association between access to green space and low mental distress [36]. Due to this finding, we believe it is expedient to discuss mediation - “if the difference method with logistic regression indicates the presence of a mediated effect, then there is in fact evidence for a mediated effect” [36].

When an association is observed between two variables A and B, there are in general three possibilities regarding causation [36]:

1. A is the cause of B.
2. B is the cause of A.
3. There is a third, underlying cause, C, which is the cause of both A and B.

Although correlation does not imply causation, the opposite is true; causation implies association. Thus, if there are theoretical reasons to expect a particular causal pathway, an observed association may be considered to support such a hypothesis.

Longitudinal studies are considered the gold standard when mediation is concerned, while cross-sectional studies may be considered unsuitable in this context, based on the trivial insight that the cause must occur before the effect. In the case of mediation, the mediator must occur before the effect, cf. Vanderweele [36] (p26). However, the framework of Baron & Kenny [33] is still used to examine the possibility of mediation in cross-sectional data [5, 37, 38] as well as in longitudinal studies [38].

Even in a longitudinal design, the data only show the order by which data have been collected, not necessarily the causal order. Independently of the study design we must know the causal order, and we cannot use the data to prove this order. Thus, if we have additional knowledge about the causal order, which is always a necessary pre-requisite for mediation, the study design is not decisive per se.

The longitudinal study of Ohrnberger et al. [26] demonstrated that there is stronger direct effect of physical health on mental health (0.24) than mental health on physical health (0.04). This is consistent with our findings. However, their study design, and particularly their mediation analysis, was different than our design, and includes several variables not included in our survey.

### Our interpretation seen in the light of literature

Our finding of correlation between access to green space and physical activity does not support the findings of Maas et al. [24], who found no such correlation. Moreover, with low mental distress as outcome, we found an interaction effect between access to green space and physical activity (only presented in the Supplementary tables B1-B2). This latter finding is in line with the findings of Astell-Burt et al. [25]. This might imply that some of the association between access to green space and low mental distress in our study may be mediated by physical activity, which contradicts their conclusion that the association between access to green space and health is not related to physical activity. However, we found no interaction effect between access to green space and physical activity when general health was the outcome, and here we agree with the findings of Maas et al. Our findings are also partially in line with the theoretical framework of Lachowycz & Jones [23], in which physical activity mediates the association between access to nature and health outcomes.

The association between access to green space and low mental distress was significantly attenuated by self-reported general health (Table 3, Model 3). However, with self-reported general health as an outcome, low mental distress did not attenuate the corresponding association (Table 3, Model 3). This demonstrates that low mental distress did not mediate the association between access to green space and general health in our material. However, this is in contrast with the findings of Dadvand et al. [37], where the association between access to green space and general health seemed to be mediated by mental health. de Vries et al. [5] and Triguero-Mas et al. [39] both found that access to green space was associated with both general and mental health, but the findings of de Vries et al. suggested that physical activity was not a mediator unless the activity took place in green surroundings. Triguero-Mas et al. concur that physical activity may not be the explaining factor for these associations.

Access to green space is regarded as a protective factor for good health, both physically and mentally [2, 6, 7, 22, 40]. Thus, concerning the associations between access to green space and the two health outcomes (mental/general health), it seems reasonable to argue that the causal pathways are mainly from access to green space towards both health outcomes, though literature demonstrates that there may be selection effects in the opposite direction;

1. “healthier people tend to choose to live in greener places” [22].
2. “(people with) higher incomes tend to live in greener areas” [24].

However, Weimann et al. show that older adults more rarely move from where they live than younger adults [41], which suggests that self-selection may not be of great importance in our case.

In our study, most people (90.7%) reported that they had access to green space, which may limit the relevance of selection hypothesis 1 (above). However, a significantly higher fraction of men than women reported good access to green space, 92.1% vs. 89.2%. In Sjögren & Stjernberg’s study, a similar gender difference was explained by income differences. They also discussed gender differences in how and whether older adults exercise outdoors, and listed economic insecurity, living alone, and fear of falling as some of the reasons why fewer women than men took part in such activity, while men more often had access to a car and used it to get to suitable areas [20]. This may also be the case in our study, but we have no data on car ownership.

**Fig. 1:**
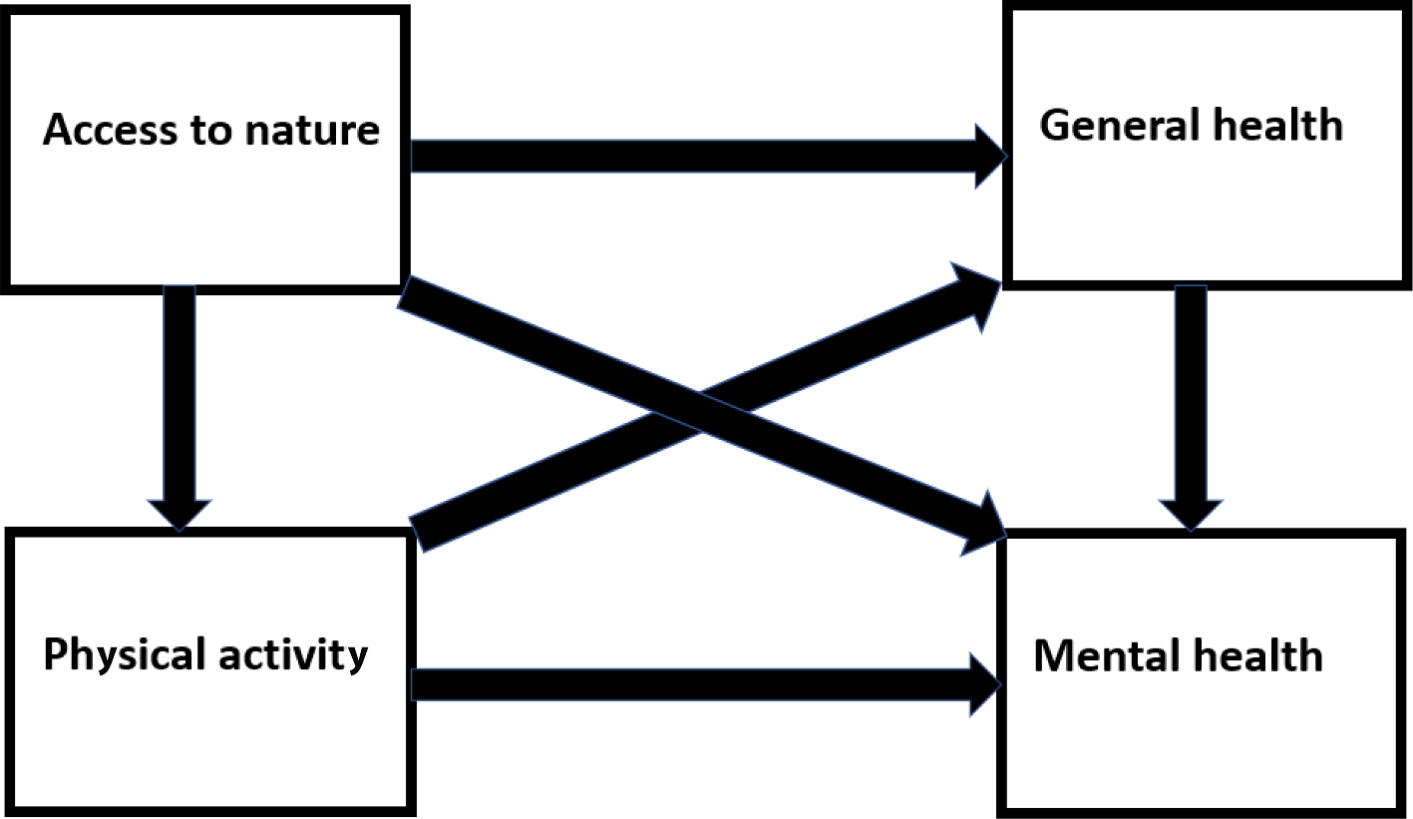
Directed Acyclic Graph (DAG) showing possible paths from access to nature and physical activity to health outcomes

Among those with higher education in our study sample (secondary school and above), a significantly higher proportion reported good access to green space than among those with lower education, 93.3% vs. 88.1%. We accounted for this by adjusting for education in the analyses. In Fig. 1 we show some possible paths from access to nature and physical activity to the health outcomes good mental health and self-reported general health.

It is possible that people who are already in good health may choose to live where they have better access to green space. It may also be possible that people who report being disabled (43% of our study population) do not consider nearby parks or nature paths accessible, though they are geographically close. However, the disability variable seemed only to a moderate degree to affect the associations between access to green space and health outcomes. More specifically, with low mental distress as outcome, including (lack of) functional disability as a confounder reduced the odds ratio of access to green space from 4.08** to 2.70**. With general health as outcome, including (lack of) functional disability as a confounder reduced the odds ratio of access to green space from 8.36** to 5.03**. In other words, our unusually large ORs may at least partially be due to marginalization effects. Self-reporting access to green space (rather than measuring the distance from where they live) may also partially explain the ORs.

A point that could be raised is whether Norway could be a special case, considering the Norwegian attitude towards “friluftsliv” [12–14, 42] would our findings be valid in other countries? We found literature from several other countries where outdoor physical activity was deemed to be important for older adults, including a study of 18 countries [6, 17–21], as well as articles postulating theoretical pathways from access to green space to health outcomes for many age groups [22–24], which suggests that Norway should not be regarded as a special case.

Kurtze et al.’s report [42], using data from two independent Norwegian questionnaire surveys from 2005 to 2007, demonstrated that in older adults (aged 65 and over), physical activity was strongly associated with general health, whereas physical activity was only associated with mental health in the younger age groups. Physical activity seemed to be of increasing importance for general health with advancing age. Outdoor activity was shown to be strongly associated with general health, but not with (lack of) serious illness in older adults. With increasing age, general health worsened, while mental health did not change in this way. Our logistic analyses (Tables 2 and 3), seem to support these findings, that physical activity is more important for general health than for mental health in older adults. We found that physical activity was associated with low mental distress, but this association seemed to a large degree to be mediated by general health. However, in the linear analyses (Supplementary Table B3), physical activity was only borderline significantly associated with general health when all the other factors were taken into account.

Næss and Hansen [34], based on hypotheses that love of nature and activity in nature lead to increased quality of life, used a large cross-sectional survey and panel data from two interviews five years apart, both representative for the Norwegian population. Among other findings, they found that exercising outdoors at least once a week was weakly associated with higher life satisfaction, particularly in people who appreciated nature. When adjusting for high activity level in general, which includes all sorts of activities, this association was no longer significant. Næss and Hansen found that being active, which includes outdoors exercise, is good for life satisfaction, which is an important component of mental health. Næss and Hansen stressed that causality was not proven and believed that loving nature and being active in nature may strengthen mental wellbeing, while mental wellbeing may increase the probability of using and appreciating nature. A selection hypothesis may be appropriate.

The idea that physical activity in nature is a panacea is thus not confirmed, which fits our findings. Our findings suggest that access to green space influences both physical and mental health, directly and indirectly. An interaction analysis is shown in Supplementary Tables B1-B4, demonstrating that there was interaction between the associations with physical activity and access to nature, for mental health, but not for general health. A simple theoretical framework for the association between access to green space via physical activity to mental health is laid out in Markevych et al. [22].

The review by Markevych et al. [22] summed up a great deal of the research on green space and health. They underscored that the literature is contradictory, with findings both of positive, neutral, and negative effects from access to green space on health outcomes. Markevych et al. explored the possible causal pathways between access to green space and health and described three domains where the effects of access to green space on health may take place: reducing harm, restoring capacities, and building capacities. Physical activity was primarily described under the third domain. Our findings are consistent with this, but we have also found that the association between access to green space and general health does not seem to go via physical activity, and we do not have the data to explore the other potential pathways described by Markevych et al.

Lachowycz & Jones [23] took up the thread from Markevych and underlined the need to expand the causal model with mediators and moderators to achieve better understanding of the pathways between access to green space and physical and mental health. The moderators listed are demographic and living context, both of which have been approached in the present article, as well as characteristics of the green space itself and the climate. We do not have data on the latter two areas. Lachowycz & Jones described the need for longitudinal studies to understand the causal pathways better, but in their grudging acceptance of cross-sectional studies also shared our pragmatic approach to data availability. We see the present article as a contribution to the debate about causative pathways between green space and positive health outcomes and believe we may have been able to demonstrate some of the mediator effects that Lachowycz et al. called for.

In a study from the Netherlands, Maas et al. [24] postulated that physical activity might be a mechanism that would lead from access to green space to self-perceived health. However, their study showed that people living near green space actually spent less time walking and bicycling for leisure, though they spent more time gardening and cycling to work. They explained the first part of this finding by the longer distances to shops etc. for people living in the greener living environment, so people chose to go by car. In more urban areas, walking and cycling were easier choices. Their study showed that access to green space had a direct effect on general health, and that this effect did not seem to go via physical activity. This corresponds well with our findings, where access to green space was strongly associated with general health in all models, without interaction with physical activity. Both of these findings seem to weaken the possible arrow between access to green space and physical activity shown in Fig. 1. The study of Maas et al. also emphasized that the environment where the study originated, in this case the Netherlands, where there is a strong tradition of bicycle commuting and a network of bicycle paths, must be included in the analysis.

### Strengths and weaknesses of our study

The participating older adults in this study may not be representative for the entire population of older adults. Our study population was randomly selected from three of Norway’s at that time 19 counties, representing a relatively sizable proportion of the Norwegian population. As in any study, our survey population may be selected, and as is often the case, be more highly educated and healthier than the general population. We have attempted to adjust for selection by adjusting for socio-economic variables. Whether there is a selection bias or not, the associations between the variables are not necessarily affected.

A proportion of the respondents were excluded from the analyses due to missing values, and only participants who responded to all questions were part of the final analyses. The following groups were slightly over-represented in the analysed population: men (original respondents 48.1% (CI 46.3-49.9%); analysed sample 51.5% (CI 50.4-52.6%), younger older adults (67–75 years); original respondents 62.4% (CI 60.7-64.1%); analysed sample 67.4% (CI 65.4-69.4%), and people with secondary education or more (original respondents 80.7% (CI 79.1 − 82.3%); analysed sample 83.3% (CI 81.7-84.9%). This demonstrates that there was a degree of selection. Despite this, since the population included in our analysis consisted of as much as 2068 individuals, we considered this population large enough not to require imputation.

One could argue that the Norwegian population may be a special case, because of the national emphasis on “friluftsliv” and generally good access to nature, since Norway has a relatively small population for the size of the country. However, there is broad agreement in the international literature about the importance of going outside and getting exercise.

It is commonly held that it is not possible to show mediation in a cross-sectional study such as ours. While we agree that a longitudinal design might have been more suited, we believe our additional arguments, including the references to Markevych et al. [22], Maas et al. [24] and Lachowycz & Jones [23], strengthen the mediation hypothesis.

Economic security shows a different dimension than income and has been shown to be strongly associated with self-rated health in older adults [39]. Economic insufficiency has been found to be a significant predictor for bad self-rated physical and mental health [38]. We chose to use this indicator instead of income, because we believe that the size of the pension, which is the only income the overwhelming majority of these study participants receive, is less important than knowing whether they feel economically secure or not and consider this a strength.

Dichotomization may lead to a loss of information. We have analysed both continuous (see Supplementary Tables B1-B4) and dichotomized variables, and though the demonstrated tendencies mostly are clearer and more easily interpreted for the dichotomized variables, all tendencies run in the same direction.

Using self-reported instead of clinical measures of general and mental health may be seen as problematic, though literature has shown that self-reported measures are valid [28, 30]. The simple frequency measure of physical activity may also be less accurate than use of a more detailed questionnaire and objective measurements, such as activity monitors. Nevertheless, such a simple measure divides the older adults in our study population into people who are physically active and those who are not. Using self-reported data is a feasible and cost-effective way of collecting data from large groups.

Regarding our question about access to green space, the high reported prevalence, approximately 90%, may be due to selection. However, it is worth bearing in mind that Norway is a country with a lot of green space, and the majority of Norwegians (of all ages) have good access to recreational areas and areas for recreational walking according to geo-data [43].

Markevych [22] describes the problems related to physical measurements of distance to green space, and that these physical measurements do not necessarily say anything about the quality of these green spaces as potential areas for outdoor recreation. Physical distance may not necessarily have anything to do with how easy it is for an individual to get out and utilize these green spaces. Markevych’s Table 1 [22] suggests that individual behavioural and perceptual measures should be considered.

Using a shorter form of the acclaimed HSCL may be problematic, but evidence from literature shows that the short form used is well established as well as very highly correlated with the original [31].

## Conclusions

In the older adults participating in this study, the association between access to green space and low mental distress appeared to be mediated by general (physical) health. The pathway from access to green space to general and mental health is complicated, and merits further study.

Physical health seems to be very important for mental health. Studies of physical activity and/or access to green space and mental health in older adults need to include physical health.

Longitudinal studies using the same questions directed to the same population cohort over time, and using other, better measures, would be a fruitful continuation of the discussion and may strengthen or weaken our hypotheses about associations between access to green space, physical activity, and health outcomes. Ensuring access to green space would in any case be beneficial for both mental and general health in older adults.

### Electronic supplementary material

Below is the link to the electronic supplementary material.

Supplementary Table A

Supplementary Tables B1-B4

## Data Availability

The datasets supporting the conclusions of this article are not publicly available, but are available upon request from the Norwegian Institute of Public Health (NIPH) and after permission from the county councils of Vestfold, Aust-Agder and Vest-Agder (as of 2020 combined into Agder county). Researchers can apply for access to the survey data here: https://www.fhi.no/en/more/access-to-data/, direct contact e-mail is datatilgang@fhi.no. Extra restrictions apply to the availability of the data with variables from national registries that require permission from the registry owners and the Norwegian Data Protectorate.
