# Supplementary material for: The association of access to green space with low mental distress and general health in older adults: a cross-sectional study
Source: BMC Geriatr. 2024 Apr 10;24:329. doi: 10.1186/s12877-024-04738-3 (PMC11007904; doi:10.1186/s12877-024-04738-3)
Supplement: Supplementary file 1 — Supplementary Table A [file 12877_2024_4738_MOESM1_ESM.docx]

# The association of access to green space with low mental distress and general health in older adults: a cross-sectional study

**Authors**: Heidi Lyshol, Rune Johansen

### Supplementary Table A. Study population by age group and proportion that responded by post, electronic questionnaire and interview

| Age group | 65-74 | | | 75+ | | | Total | | |
| --- | --- | --- | --- | --- | --- | --- | --- | --- | --- |
|  | Men | Women | Total | Men | Women | Total | Men | Women | Total |
| Post | 598 (80.2%) | 564 (86.8%) | 1 162 (83.2%) | 283 (88.7%) | 320 (90.4%) | 603 (89.6%) | 881 (82.8%) | 884 (88.0%) | 1 765 (85.3%) |
| Web | 106 (14.2%) | 55 (8.5%) | 161 (11.5%) | 21 (6.6%) | 14 (4.0%) | 35 (5.2%) | 127 (11.9%) | 69 (6.9%) | 196 (9.5%) |
| Phone | 41 (5.6%) | 31 (4.7%) | 72 (5.3%) | 15 (4.7%) | 20 (5.6%) | 35 (5.2%) | 56 (5.3%) | 51 (5.1%) | 107 (5.2%) |
| Total | 745 | 650 | 1 395 | 319 | 354 | 673 | 1 064 | 1 004 | 2 068 |
