# Supplementary material for: The association of access to green space with low mental distress and general health in older adults: a cross-sectional study
Source: BMC Geriatr. 2024 Apr 10;24:329. doi: 10.1186/s12877-024-04738-3 (PMC11007904; doi:10.1186/s12877-024-04738-3)
Supplement: Supplementary file 2 — Supplementary Tables B1-B4 [file 12877_2024_4738_MOESM2_ESM.docx]

# The association of access to green space with low mental distress and general health in older adults: a cross-sectional study

**Authors**: Heidi Lyshol, Rune Johansen

### Supplementary Table B1. Model 2. Linear regression. Dependent variable HSCL-5 score (low mental distress). Excluding general health. Including interaction terms.^1^

| Variable | Standardized $\beta$ | B | 95% CI |
| --- | --- | --- | --- |
| Access to green space | 0.517* | 0.780 | 0.139, 1.421 |
| Physical activity | 0.418** | 0.131 | 0.053, 0.208 |
| General health | - | - | - |
| Access to culture/sports venues | 0.086** | 0.126 | 0.064, 0.188 |
| Economic security | 0.127** | 0.219 | 0.148, 0.290 |
| Lack of functional disability | 0.187** | 0.169 | 0.130, 0.207 |
| Sex | 0.028 | 0.025 | -0.116, 0.166 |
| Age | 0.088 | 0.006 | -0.003, 0.016 |
| Education | 0.167 | 0.046 | -0.003, 0.095 |
| Access to green space X physical activity | 0.381** | 0.056 | 0.015, 0.096 |
| Access to green space X Sex | 0.021 | 0.013 | -0.111, 0.137 |
| Access to green space X Age | -0.454* | -0.008 | -0.016, 0.000 |
| Access to green space X Education | 0.169 | 0.038 | -0.006, 0.082 |

** Significant at 1% level
*Significant at 5% level
^1^ Adjusted explained variance 11.8%

Supplementary Table B2. Model 3. Linear regression. Dependent variable HSCL-5 score (low mental distress). Including general health as a predictor and interaction terms.^1^

| Variable | Standardized $\beta$ | B | 95% CI |
| --- | --- | --- | --- |
| Access to green space | 0.318 | 0.481 | -0.132, 1.095 |
| Physical activity | 0.319** | 0.099 | 0.025, 0.174 |
| General health | 0.325** | 0.175 | 0.151, 0.200 |
| Access to culture/sports venues | 0.081** | 0.118 | 0.059, 0.178 |
| Economic security | 0.098** | 0.169 | 0.101, 0.238 |
| Lack of functional disability | 0.079** | 0.071 | 0.032, 0.111 |
| Sex | 0.077 | 0.068 | -0.068, 0.203 |
| Age | 0.032 | 0.002 | -0.007, 0.011 |
| Education | 0.112 | 0.031 | -0.016, 0.078 |
| Access to green space X physical activity | 0.333* | 0.048 | 0.010, 0.087 |
| Access to green space X Sex | -0.037 | -0.022 | -0.142, 0.098 |
| Access to green space X Age | -0.261 | -0.005 | -0.012, 0.003 |
| Access to green space X Education | 0.148 | 0.033 | -0.009, 0.075 |

** Significant at 1% level
*Significant at 5% level
^1^ Adjusted explained variance 19.3%

Supplementary Table B3. Model 2. Linear regression. Dependent variable general health. Excluding HSCL. Including interaction effects.^1^

| Variable | Standardized $\beta$ | B | 95% CI |
| --- | --- | --- | --- |
| Access to green space | 0.593** | 1.678 | 0.622, 2.733 |
| Physical activity | 0.203(*) | 0.118 | -0.008, 0.244 |
| HSCL-5 score (low mental distress) | - | - | - |
| Access to culture/sports venues | 0.036(*) | 0.098 | -0.003, 0.199 |
| Economic security | 0.093** | 0.292 | 0.179, 0.405 |
| Lack of functional disability | 0.338** | 0.566 | 0.504, 0.628 |
| Sex | -0.021 | -0.034 | -0.264, 0.197 |
| Age | 0.165** | 0.021 | 0.006, 0.036 |
| Education | 0.225** | 0.116 | 0.036, 0.196 |
| Access to green space X physical activity | 0.052 | 0.014 | -0.052, 0.080 |
| Access to green space X Sex | -0.024 | -0.026 | -0.230, 0.178 |
| Access to green space X Age | -0.535** | -0.017 | -0.030, -0.005 |
| Access to green space X Education | 0.141 | 0.059 | -0.013, 0.131 |

** Significant at 1% level
* Significant at 5% level
 (*) Boundary significant at 5% level
^1^ Adjusted explained variance 27.6%

Supplementary Table B4. Model 3. Linear regression. Dependent variable general health. Including HSCL score as a predictor and interaction terms.^1^

| Variable | Standardized $\beta$ | B | 95% CI |
| --- | --- | --- | --- |
| Access to green space | 0.445* | 1.250 | 0.221, 2.378 |
| Physical activity | 0.089 | 0.051 | -0.176, 0.073 |
| HSCL-5 score (low mental distress) | 0.266** | 0.493 | 0.423, 0.562 |
| Access to culture/sports venues | 0.012 | 0.032 | -0.068, 0.132 |
| Economic security | 0.056** | 0.180 | 0.065, 0.295 |
| Lack of functional disability | 0.282** | 0.471 | 0.408, 0.533 |
| Sex | -0.031 | -0.051 | -0.278, 0.171 |
| Age | 0.136* | 0.017 | 0.002, 0.032 |
| Education | 0.180* | 0.092 | 0.013, 0.171 |
| Access to green space X physical activity | -0.060 | -0.016 | -0.081, 0.049 |
| Access to green space X Sex | -0.010 | -0.011 | -0.212, 0.189 |
| Access to green space X Age | -0.407** | -0.013 | -0.025, -0.001 |
| Access to green space X Education | 0.087 | 0.036 | -0.035, 0.107 |

** Significant at 1% level
* Significant at 5% level
^1^ Adjusted explained variance 34.0%
